# Editorial: Volume 2: real world evidence (RWE): paths to enhancing patient access to new medications

**DOI:** 10.3389/jpps.2026.16236

**Published:** 2026-03-04

**Authors:** Allison Wills, Catherine Y. Lau

**Affiliations:** 1 20Sense Corp., Toronto, ON, Canada; 2 Consultant, Toronto, ON, Canada

**Keywords:** Canada, drugs, FAST, pCPA, reimbursement, market access, patient access

## NEW PATHWAYS, OLD PROBLEMS: IS PATIENT ACCESS TO NEW MEDICATIONS IMPROVING IN CANADA?

Timely and equitable access to innovative medicines is the cornerstone for improving patient health outcomes and the overall quality of healthcare. Canada’s public reimbursement system for new medicines is widely recognized as complex and challenging, and Canada ranks among the slowest Organization for Economic Co-operation and Development (OECD) countries for patient access to novel medications, taking more than 1.5 years after regulatory approval for a new drug to be listed on the public plans in Canada [1]. This slow pace is recognized as a result of the multi-layered drug reimbursement processes involving federal, pan-Canadian, and provincial reviews, which are frequently opaque and difficult to navigate, and cause significant delays in access to urgently-needed treatments [2, 3].

Health Canada does not significantly contribute to these delays, as it has modernized its regulatory processes and positioned itself among the top tier international review agencies, following on the footsteps of leading regulatory agencies such as FDA and EMA [4]. Health Canada’s three pathways for drug approvals – Standard Reviews, Priority Reviews and Notice of Compliance with Conditions (NOC/c) – provide flexible options for the submission of new medicines, including those supported by preliminary evidence, treatments for rare (orphan) conditions, and drugs with exceptional efficacy [5]. Health Canada also was among the first regulators to participate in Project Orbis [6], an FDA-led international joint review initiative for promising oncology drugs, underscoring its reputation as a science-driven, evidence-based regulator.

So where does the slowdown come from? After Health Canada grants regulatory approvals, approximately half of drugs [7] face additional scrutiny through health technology assessments (HTA) by Canada’s Drug Agency (CDA-AMC) or Quebec’s Institut national d’excellence en santé et en services sociaux (INESSS), followed by price negotiations with the pan-Canadian Pharmaceutical Alliance (pCPA) and, finally, each provincial jurisdiction independently decides whether to include the medication on its public drug formulary [8]. These steps can stretch out the time between approval and actual patient access, leading to inconsistent availability of medications across different regions [9]. The typically sequential HTA, pCPA and provincial formulary processes have been identified as main contributors to delays in patient access.

## Recent reforms to accelerate access: early impact and remaining gaps

From 2023 to 2025, Canada introduced three new processes to accelerate access to novel medicines [10, 11]. (Other processes, such as Pharmaceuticals with Anticipated Comparable Efficacy and Safety (PACES) and Targeted Negotiation Process (TNP), are not being mentioned here as they are not pertinent to novel medicines). These are:

- TLR-pTAP: Canada’s Drug Agency’s Time-limited recommendation (TLR) category, which established a mechanism to provide time-limited HTA recommendations to drugs with evidence uncertainties approved under NOC/c, and the corresponding pan-Canadian Pharmaceutical Alliance’s Temporary Access Process (pTAP), were introduced in September 2023 [12, 13].
- pCPA ENP: The pan-Canadian Pharmaceutical Alliance’s (pCPA) Early Negotiation Process (ENP), introduced in October 2025, provides an expedited negotiation pathway for cancer drugs that are part of Project Orbis, whereby the negotiation process begins when a HTA body accepts a submission [14].
- Ontario FAST: Also in October 2025, Ontario launched the Funding Accelerated for Specific Treatments (FAST) 3-year pilot program for selective oncology drugs approved by the Orbis process, and provides early public funding while the pCPA and drug manufacturer negotiate the drug price [15].

These processes enable concurrent reviews by 2-3 agencies – an approach already adopted for more than a decade by various European agencies – and signal the emergence of an important continuous improvement mindset across organizations as well as a convergence of institutional silos. However, it remains uncertain whether they will lead to a tangible reduction in delays to drug access. Early results from the TLR-pTAP processes suggest that the stringent criteria for acceptance into these pathways have limited uptake [16]. As the pCPA’s ENP and Ontario’s FAST programs have only recently launched, more time will be required to analyse their impact.

## Proposed strategies towards tangible timely access solutions

- Continuing to prioritize operational efficiencies at the pCPA: Recent performance improvements at the pCPA illustrate how strengthening core operational timelines can yield meaningful access gains at scale. Total negotiation times across all drugs declined from approximately 11 months in 2020 to 6.5 months in 2025, representing a 41% reduction, with similar improvements observed for oncology therapies [17]. Although less conspicuous than newly introduced pathway initiatives, these efficiency gains affect the largest number of medicines and directly shorten the time to patient access. Establishing and maintaining a reliable baseline performance at the pCPA can create the capacity needed to address more complex files, using targeted pathways and innovative approaches such as outcomes-based agreements or other innovative access models requiring real-world data to address remaining uncertainties.
- Protected funding for innovation: Building on the Ontario FAST program, it is critical to further establish Canada-wide dedicated funding mechanisms to support innovative and equitable reimbursement strategies, especially for high-cost and rare disease therapies. The National Strategy for Drugs for Rare Diseases, which launched in 2023, has “yet to translate into improvements in access to treatments for patients with rare diseases” [18].
- Continuous policy and process evolution: Guidelines and policies for enabling timely public access to innovative medicines need to align with advancements in medical practice and emerging real-world evidence. This includes, for example, broadening the scope of accelerated reimbursement pathways to include a broader range of high-priority therapies. Currently, the three paths described in this paper focus solely or primarily on oncology.
- Establishing a pathway for joint pricing models for high-cost therapies and drugs for rare diseases: Proactive collaboration between pharmaceutical companies, HTA agencies, the pCPA and public payers will enable the development realistic, value-based pricing models that link reimbursement to real-world clinical outcomes for high-cost treatments such as CAR-T, gene therapies, and other drugs for rare diseases, tailored to the Canadian funding environment.

## Conclusion: moving toward timelier access, slowly but surely

The introduction of novel pathways brings hope to Canadian patients that “more equitable access to life-saving treatments” can be achieved [19]. Although these approaches have led to some improvements in patient access, significant hurdles persist. In addition to the challenges noted above, gaps in health-system readiness for the implementation of new drugs have recently emerged as a potential obstacle [20]. The growing influence of artificial intelligence in drug discovery and development is expected to transform therapies entering the market, which may further strain current access pathways. As such, those championing the early, timely and equitable availability of new medicines in Canada must balance forward-thinking ambition with pragmatic solutions, recognizing the complex relationships between government agencies, pharmaceutical companies, and patient communities.

Post Submission Note: It was announced on January 22, 2026, that since October 2025 six cancer drugs have been accelerated for funding through the FAST pilot [21].
